# Meta‐Analysis to Determine Optimal Timing for Repair of Inguinal Hernia in Pre‐Term Infants

**DOI:** 10.1002/wjs.70014

**Published:** 2025-07-25

**Authors:** Tallal Mushtaq Hashmi, Hadiah Ashraf, Tehreem Fatima, Amna Javed, Abdullah, Muhammad Abdullah Kashif, Soban Raza, Khawaja Haris Ismail, Arooj Fatima, Javed Iqbal, Raheel Ahmed, Areeba Ahsan, Mushood Ahmed

**Affiliations:** ^1^ Rawalpindi Medical University Rawalpindi Pakistan; ^2^ King Edward Medical University Lahore Pakistan; ^3^ Foundation University Medical College Islamabad Pakistan; ^4^ CMH Kharian Medical College Kharian Pakistan; ^5^ Hamad Medical Corporation Doha Qatar; ^6^ Imperial College London London UK; ^7^ Royal Brompton Hospital London UK

## Abstract

**Background:**

Studies have shown conflicting findings regarding the optimal time for the repair of inguinal hernia in preterm infants. Our review aims to comprehensively synthesize the available evidence and provide robust, up‐to‐date insights into the clinical outcomes of delayed versus early repair of inguinal hernia in these patients.

**Methods:**

A comprehensive search across PubMed, Cochrane Library, Clinicaltrials.gov, and Embase was conducted from inception until July 2024 to include RCTs and cohort studies comparing early versus delayed inguinal hernia repair. The odds ratios (ORs) were pooled along with corresponding 95% confidence intervals (CIs) for all clinical endpoints with the random effects model using RevMan 5.4.

**Results:**

Our meta‐analysis pooled 10 studies involving 4253 preterm infants. The early hernia repair group had lower odds of developing incarceration (OR 0.43, 95% CI 0.34–0.54, *p* < 0.00001) but increased respiratory complications (OR 5.17, 95% CI 3.97–6.73, *p* < 0.00001) and recurrence rates (OR 3.25, 95% CI 1.18–8.92, *p* = 0.02) as compared to the delayed group. There were no statistically significant differences in testicular atrophy (OR 1.13, *p* = 0.91), iatrogenic ascending testis (OR 1.71, *p* = 0.61), postoperative hydrocele (OR 3.69, *p* = 0.21) or bowel injury (OR 1.16, *p* = 0.88) between early and delayed repair groups.

**Conclusion:**

Timing of inguinal hernia repair requires balancing the risk of incarceration with anesthetic and recurrence concerns. Early hernia repair reduces incarceration but is associated with higher rates of respiratory complications and recurrence.

## Graphical abstract

Studies have shown conflicting findings regarding the optimal time for the repair of inguinal hernia in preterm infants. Our review aims to comprehensively synthesize the available evidence and provide robust, up‐to‐date insights into the clinical outcomes of delayed versus early repair of inguinal hernia in these patients. A comprehensive search across PubMed, Cochrane Library, Clinicaltrials.gov, and Embase was conducted from inception until July 2024 to include RCTs and cohort studies comparing early versus delayed inguinal hernia repair.

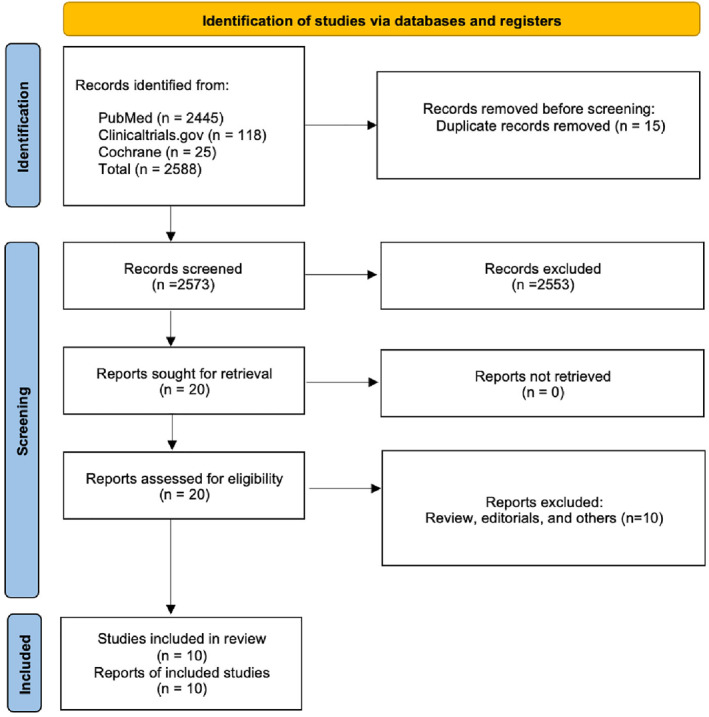

## 1 Introduction

One of the most prevalent pediatric surgical disorders is inguinal hernia. Incidences of inguinal hernia in full‐term and prematurely delivered neonates vary from 1% to 5% and 30%, respectively. Male‐to‐female incidence ratios range from 4 to 10:1 [1]. Inguinal hernias are more common in preterm neonates and the risk rises with decreasing gestational age. There is uncertainty over the optimal time for surgical repair in this group due to comorbid conditions [2].

Preterm newborns are considered high‐risk patients due to their underlying medical conditions. The most common morbidities include anemia, congenital heart disease, intraventricular hemorrhage, bronchopulmonary dysplasia, necrotizing enterocolitis, respiratory distress syndrome, and patent ductus arteriosus. Preterm infants typically have a thin‐layered, edematous hernia sac, which requires a skillful and experienced surgeon to dissect because opening it unintentionally increases the chance of recurrence. According to pertinent bibliographic data, preterm newborns have greater rates of hernia recurrence, ranging from 5% to 14.1% [3].

Since there is a chance of incarceration, strangulation, testicular shrinkage, and patient loss to follow‐up and care access, early repair of inguinal hernia is frequently advised, particularly after newborn admission in the neonatal intensive care unit (NICU) [4]. Delaying surgery for children with inguinal hernias carries a significant risk of incarceration [5]. On the other hand, Bawazir et al. reported that in the early repair group, there was an increased risk of testicular atrophy, admission to the pediatric intensive care unit (PICU), and recurrence of inguinal hernia after surgery [6]. Moreover, it was found that infants in the late repair group spent less time in the NICU and spent fewer days in the hospital after surgery. Infants with bronchopulmonary dysplasia and those whose gestational age was less than 28 weeks benefited most after late repair. The trial conducted by Blakeley further supported this idea of postponing hernia treatment, indicating a 97% chance that children who had a surgical repair performed later on would have fewer adverse outcomes. Additionally, researchers discovered that over 10% of the hernias in the delayed group resolved without any surgical intervention [7]. Some studies advise early repair regardless of gestational age or birth weight; others contend that infants born at earlier gestational ages or with lower birth weights may benefit from delayed repair [8].

With conflicting results, several studies have looked into the best time to repair an inguinal hernia in preterm newborns. According to a 2005 poll of pediatric surgeons, 63% of surgeons said they would prefer to treat premature infants' hernias before hospital discharge, whereas 23% explicitly said they would postpone surgery longer, usually to accommodate increases in post‐conception age or weight. Therefore, the optimal time to initiate IHR in premature infants is yet unknown [4].

Our meta‐analysis, which includes 10 studies involving 4253 preterm infants, stands as the most comprehensive and current review on this subject, incorporating findings from a recent randomized controlled trial [7].

## 2 Materials and Methods

This meta‐analysis was registered with PROSPERO (CRD42024583512) and conducted in accordance with the Cochrane Handbook for Systematic Reviews of Interventions and the Preferred Reporting Items for Systematic Reviews and Meta‐Analyses (PRISMA) (Supporting Information S1: Table S1) guidelines [9, 10]. As the analysis did not involve direct collection or investigation of patient data by the authors, formal ethical approval was not required.

### 2.1 Data Sources and Searches

**FIGURE 1:**
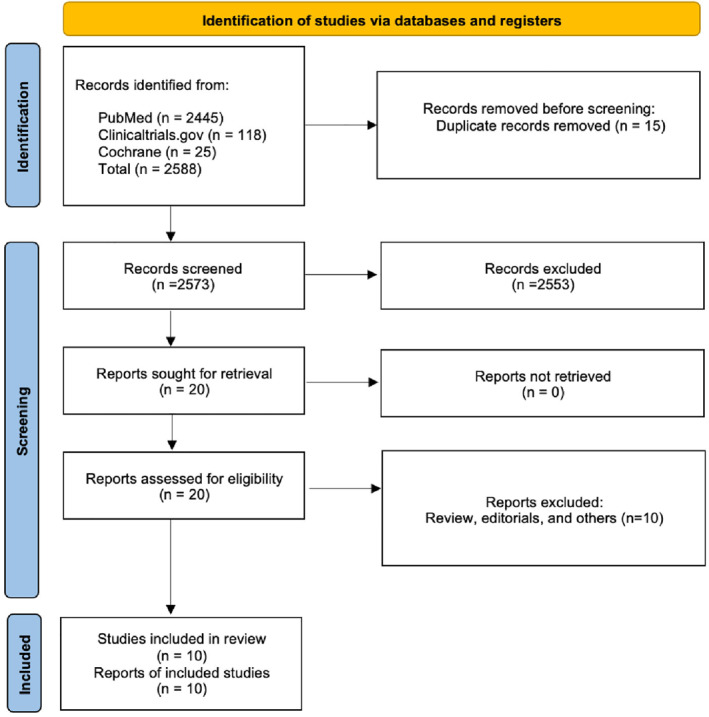
PRISMA flowchart showing the study selection process.

A comprehensive literature search was performed using PubMed, the Cochrane Library, clinicaltrials.gov, and Embase, covering all records from inception until July 2024 (Figure 1). There were no language restrictions. The search strategy included the following keywords and their MeSH terms: (“Preterm Infants” OR “Premature Infant” OR “Preterm Neonates” OR “Premature neonates”) AND (“Herniorrhaphy” OR “Hernia repair” OR “Inguinal hernia” OR “Inguinal Hernia Repair” OR “Early Repair” OR “Immediate Repair” OR “Early Surgery” OR “Immediate Surgery” OR “before NICU discharge”) AND (“Delayed Repair” OR “Delayed Surgery” OR “Late Repair” OR “Late Surgery” OR “after NICU discharge”) (Supporting Information S1: Table S2).

### 2.2 Study Selection

Articles identified through the literature search were imported into EndNote 21, where TMH removed duplicates. The remaining articles were initially screened by title and abstract, followed by a full‐text review to assess relevance. Two independent reviewers (TMH and MA) conducted both the primary and secondary screenings in a blinded manner, with disagreements resolved through discussion. Eligible studies were required to compare outcomes of early versus delayed herniotomy, involve randomized controlled trials, retrospective studies, or observational cohorts, and focus on preterm infants diagnosed with inguinal hernia during their first hospitalization. Exclusion criteria included combining data with full‐term newborns, unclear timing of diagnosis, and outcomes not separated by early versus delayed groups. Backward snowballing was also used to identify additional relevant studies.

### 2.3 Outcome Measures

The primary outcome assessed was hernia incarceration, whereas secondary outcomes included respiratory complications, recurrence, testicular atrophy, postoperative hydrocele, bowel injury, and iatrogenic ascending testis. Hernia incarceration was defined as an irreducible inguinal hernia necessitating surgical intervention. Respiratory complications were characterized as postoperative apnea, respiratory insufficiency, or the need for extended postoperative ventilatory support.

### 2.4 Data Extraction

Following the primary and secondary screenings, RCTs and cohort studies that met the inclusion criteria were selected. Two independent reviewers were involved in the study selection process, and any disagreements or uncertainties regarding inclusion or exclusion were resolved through discussion. Data on baseline characteristics and individual outcome measures were extracted from the results sections of the studies using a pre‐piloted excel spreadsheet. The extracted baseline characteristics included patient population, gestational age at birth, sex, birth weight, postnatal days at diagnosis, post‐conceptional age at surgery, and weight at surgery. The extracted outcomes encompassed the rate of hernia incarceration, spontaneous resolution, respiratory complications, postoperative reintubation, pneumothorax, apnea or hypoxemia, bradycardia, surgical complications, hydrocele, recurrence rate, bowel injury, testicular atrophy, and iatrogenic ascending testis.

### 2.5 Study Quality and Risk of Bias

The quality and risk of bias for the randomized controlled trials (RCTs) and cohort studies were assessed using the “RoB 2: A Revised Cochrane Risk‐of‐Bias Tool” for RCTs and the “Risk of Bias in Non‐Randomized Studies of Interventions (ROBINS‐I)”tool for cohort studies. Three independent reviewers evaluated the risk of bias in each study, after which the results were pooled and visualized using the Robvis tool to generate a traffic light plot (Supporting Information S1: Figures S1 and S2). Because of the inclusion of fewer than 10 studies for each outcome, publication bias was not assessed through a funnel plot analysis.

### 2.6 Data Analysis

As argued by Choo et al. the incidence of incarceration can only be accurately determined through prospective or randomized studies [11]. For observational studies, we used the same mathematical approach as used by Choo et al. to calculate the adjusted incarceration number for each group [11]. All statistical analyses were conducted using RevMan 5.4 (The Cochrane Collaboration, Copenhagen, Denmark). Pooled odds ratios (OR) were calculated for each outcome using the Mantel–Haenszel method, with significance defined as a *p*‐value of less than 0.05 and 95% confidence intervals (CIs). A random‐effects model was employed for all analyses. Heterogeneity was assessed using the *I* ^2^ statistic, where *I* ^2^ > 50% indicated substantial heterogeneity. A leave‐one‐out sensitivity analysis was performed to identify any outlier studies. Following Cochrane guidelines, publication bias was not assessed using funnel plots or statistical tests, as fewer than 10 studies were included for each outcome.

## 3 Results

### 3.1 Study Selection

The search yields a total of 2588 records. After removing 15 duplicates, 2573 records were screened based on title and abstract. Of these, 2553 were excluded, leaving 20 reports for full‐text assessment. No record was excluded at the retrieval stage. Ultimately, 10 studies were included in the review (Figure 1). The study by Crankson et al. was excluded from our analysis due to the presence of participants in the delayed group who were diagnosed with inguinal hernia post‐discharge following birth.

### 3.2 Study Characteristics

All the included studies were retrospective cohort study design except for Blakely et al., which is the recent randomized controlled trial [7, 8, 11, 12, 13, 14, 15, 16, 17, 18]. This meta‐analysis included a total of 4253 preterm infants. Out of these, 345 (10.7%) infants developed incarceration. The mean gestational age in the early group was 27.65 ± 0.73 weeks, while for the delayed group, it was 29.77 ± 2.09 weeks (Table 1). Among all the preterm infants, 3394 (80%) were male. The surgical approach, either laparoscopic or open repair, was reported only by four of the included studies.

**TABLE 1:** Characteristics of included studies.

Author, year	Study design	Sample size		GA (weeks)		Males (*n*)		Birth weight (g)^a^		Postnatal days at diagnosis^a^		Post‐conceptional age (weeks)^a^		Weight at surgery (kg)^a^	
		Early Delayed		Early Delayed		Early Delayed		Early Delayed		Early Delayed		Early Delayed		Early Delayed	
Choo et al. 2023 [11]	Ret	189	30	29.0 ± 2.99	29.83 ± 3.89	102	16	1103 (880–1390)	1256 (860–1590)	47 (28–67)	33.5 (21–61)	37.7 (36–40)	42.1 (38–49)	2.22 (2.00–2.70)	3.27 (2.21–4.60)
Cho et al. 2023 [8]	Ret	109	40	27.9 ± 2.9	31.4 ± 2.7	78	28	905.4 ± 335	1510 ± 568	Not reported	Not reported	36.6 ± 41.6	54.6 ± 55.9	2467.0 ± 623.6	5915.0 ± 2046.7
Sulkowski et al. 2015 [12]	Ret	1363	667	28 ± 4.46	28.33 ± 3.72	1157	603	977 (731–1500)	1016 (750–1468)	Not reported	Not reported	40 (38–43)	49 (44–56)	Not reported	Not reported
Takahashi et al. 2012 [13]	Ret	14	33	27.6 ± 3.1	30.2 ± 4.0	7	21	928 ± 353	1189 ± 486	36.6 ± 3.1	38.1 ± 4.4	42.2 ± 5.7	48.8 ± 3.7	2919 ± 390	4583 ± 995
Khan et al. 2018 [14]	Ret	115	148	28.01 ± 3.13	32.30 (3.63)	202	61	1070 ± 570	1750 ± 720	37.07 (7.33)	42.96 (9.96)	39.49 (3.97)	40.80 (7.36)	Not reported	Not reported
Lee et al. 2010 [15]	Ret	45	35	27.8 ± 3.2	29.4 ± 3.4	65	15	1002 ± 539	1126 ± 460	Not reported	Not reported	9.2 ± 3.5	14.7 ± 4.5	2328 ± 278	3664 ± 703
Pandey et al. 2016 [16]	Ret	23	16	26.2 ± 2.6	26.2 + 2.7	17	11	753 ± 158	744 ± 131	Not reported	Not reported	Not reported	Not reported	Not reported	Not reported
Blakely et al. 2024 [7]	RCT	159	149	27 ± 3	27 ± 3	14	133	835 (615–1072)	810 (660–1010)	Not reported	Not reported	41 (39–44)	57 (52–61)	3.1 (2.5–3.6)	5.9 (4.7–6.9)
Youn et al. 2018 [17]	Ret	18	72	27.33 ± 4.83	30.67 ± 8.32	5	59	880 (430–2140)	1.46 (0.74–3.20)	Not reported	Not reported	41.8 (35.1–48.3)	43.7 (30.7–84.0)	2.45 (1.46–4.50)	4.30 (1.91–7.80)
Sacks et al. 2023 [18]	Ret	634	394	27.67 ± 4.48	32.33 ± 4.48	542	258	830 (650–1300)	1400 (1000–2100)	Not reported	Not reported	120 (87–165)	147 (88–239)	3.9 (2.9–5.0)	5.8 (4.3–7.4)

Abbreviations: RCT, randomized controlled trial; Ret, retrospective cohort study.

^a^ Data reported as mean SD or median (IQR).

### 3.3 Primary Outcome

#### 3.3.1 Incarceration

**FIGURE 2:**
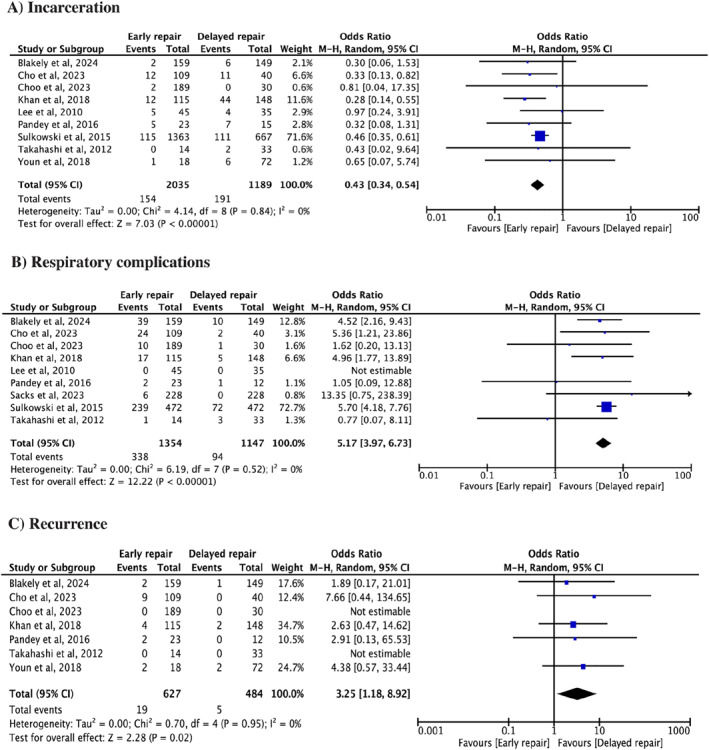
Forest plots for (A) Incarceration, (B) Respiratory complication, and (C) Recurrence.

All included studies reported on incarceration. The pooled analysis demonstrated that the patients in the early group had lower odds of developing incarceration as compared to the delayed group (OR 0.43, 95% CI 0.34–0.54, *I* ^2^ = 0%, *p* < 0.00001) (Figure 2A).

### 3.4 Secondary Outcomes

#### 3.4.1 Respiratory Complications

Respiratory complications including apnea, postoperative reintubation, postoperative respiratory insufficiency, postoperative ventilation, and bradycardia were reported by eight studies. The pooled analysis demonstrated that patients in the early group had higher odds of developing respiratory complications as compared to the delayed group (OR 5.17, 95% CI 3.97–6.73, *I* ^2^ = 0%, *p* < 0.00001) (Figure 2B).

#### 3.4.2 Recurrence

Upon pooled analysis from eight studies, it was found that patients in the early group had higher odds of recurrence as compared to the delayed group (OR 3.25, 95% CI 1.18–8.92, *I* ^2^ = 0%, *p* = 0.02) (Figure 2C).

#### 3.4.3 Atrophic Testis

**FIGURE 3:**
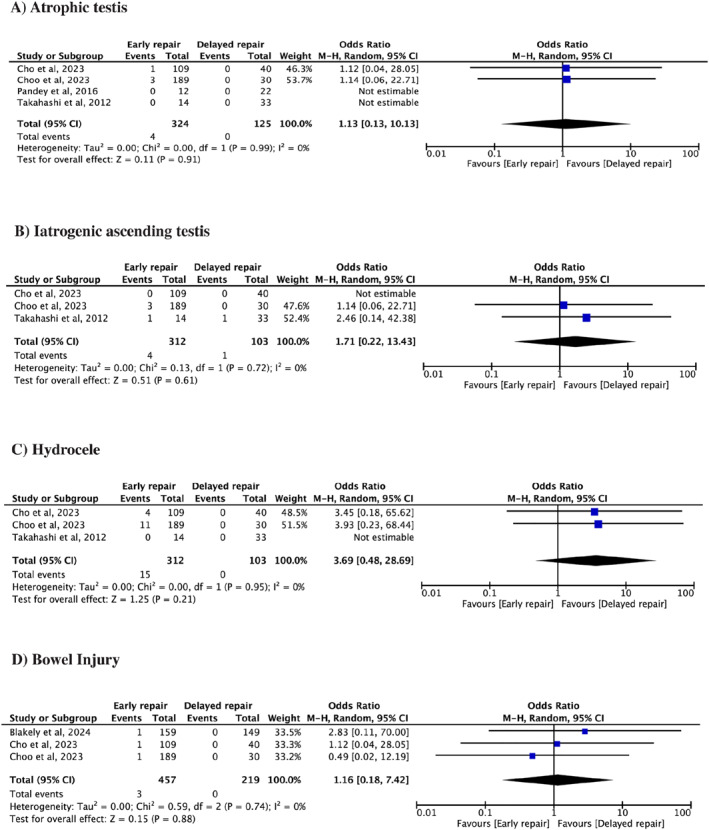
Forest plots for (A) Atrophic testes, (B) Iatrogenic ascending testis, (C) Hydrocele, and (D) Bowel injury.

Testicular atrophy was defined as loss of viability and subsequent shrinkage of the testis due to ischemia or damage from the hernia or surgical intervention. The pooled analysis from four studies showed no statistically significant difference for testicular atrophy among the two groups (OR 1.13, 95% CI 0.13–10.13, *I* ^2^ = 0%, *p* = 0.91) (Figure 3A).

#### 3.4.4 Iatrogenic Ascending Testis

The pooled analysis of three studies revealed no statistically significant difference in the odds of developing iatrogenic ascending testes between the two groups (OR 1.71, 95% CI 0.22–13.43, *I* ^2^ = 0%, *p* = 0.61) (Figure 3B).

#### 3.4.5 Hydrocele

The pooled analysis showed that the odds of developing post‐operative hydrocele were comparable between the two groups (OR 3.69, 95% CI 0.48–28.69, *I* ^2^ = 0%, *p* = 0.21) (Figure 3C).

#### 3.4.6 Bowel Injury

The pooled analysis from three studies showed a nonsignificant difference for surgical bowel injury among the two groups (OR 1.16, 95% CI 0.18–7.42, *I* ^2^ = 0%, *p* = 0.88) (Figure 3D).

## 4 Discussion

This comprehensive meta‐analysis, encompassing 10 studies and involving a total of 4253 preterm infants, represents the largest and most up‐to‐date synthesis on this topic, incorporating data from the recent randomized controlled trial [7]. Our results show that early hernia repair is associated with lower odds of incarceration. However, the odds of recurrence and respiratory complications are higher with early repair.

Our primary outcome was incarceration, which is associated with significant morbidity and mortality [19]. Our results indicated that patients in the early group had a lower likelihood of developing incarceration compared to those in the delayed group. The risk of incarceration in children with an inguinal hernia varies between 3% and 16%, with the highest incidence estimated to be 30% in premature infants [20]. Incarceration can result in bowel obstruction, ischemia, and even perforation if not promptly addressed, making early diagnosis and intervention crucial [19].

Incarceration is also associated with a higher risk of recurrence [21]. The recurrence rate for an incarcerated hernia following open repair can range from 15% to 20% [21]. Our analysis indicates a statistically significant reduction in the odds of recurrence in the delayed group relative to the early group. The early repair group also had a higher likelihood of experiencing respiratory complications. The definition of respiratory complications varied across the identified studies, yet they were collectively categorized under the term “respiratory complications.” Respiratory complications, such as apnea, postoperative reintubation, respiratory insufficiency, postoperative ventilation, and bradycardia, were reported in eight of the included studies.

The risk of developing other surgical complications including post‐operative hydrocele, iatrogenic ascending testis, and testicular atrophy, showed a statistically nonsignificant difference between the two groups. This is likely due to the limited sample size and lack of robust evidence from prospective randomized controlled trials (RCTs). Additionally, potential sources of bias, variability in study methodologies, and inconsistencies in outcome reporting may also contribute to the observed limitations.

Delaying the hernia repair to allow for spontaneous resolution is not well advocated [22]. However, this approach might enable a subset of patients to achieve spontaneous resolution. In a recent case series, one‐third of the cohort, particularly females, experienced clinical regression of their hernias upon follow‐up examination without significant morbidity [23]. Postponing surgical intervention for inguinal hernia may be a viable approach in selected stable infants, provided they are closely monitored under the careful supervision of both parents and the surgeon.

It is important to recognize the limitations of the current review. The inclusion of only one randomized controlled trial (RCT) and a limited number of retrospective studies, along with insufficient details on surgical techniques, comorbidities, anesthesia, and variable follow‐up durations, may have affected the assessment of outcomes. Additionally, we were unable to evaluate potential covariates due to the lack of individual participant data, which could have provided more nuanced insights. Variations in the definitions of early and late repair among studies are unlikely to have substantially impacted the results. However, unclear definitions of incarcerated hernia in some studies may have led to misclassification, influencing the overall findings. The observed data heterogeneity and reliance on statistical adjustments for incarceration rates underscore the need for prospective and randomized studies to deliver more accurate and reliable information.

## 5 Conclusion

The timing of inguinal hernia repair requires a careful balance between the surgical risk of incarceration, which favors early intervention, and the perioperative anesthetic risks and potential for recurrence, which may suggest delaying the procedure. Based on current evidence, delaying repair may be safe for preterm infants with respiratory issues, provided there are no hernia complications. Ultimately, the decision should be tailored to individual patient risks and clinical contexts. However, prospective studies, randomized controlled trials, and long‐term follow‐up are needed to provide more precise comparisons and inform clinical decision‐making.

## Author Contributions

**Tallal Mushtaq Hashmi:** conceptualization, data curation, formal analysis, investigation, methodology, software, validation, visualization, writing – original draft. **Hadiah Ashraf:** conceptualization, data curation, investigation, methodology, software, validation, visualization, writing – original draft. **Tehreem Fatima:** data curation, formal analysis, methodology, writing – original draft, writing – review and editing. **Amna Javed:** investigation, methodology, resources, validation, visualization, writing – original draft. **Abdullah:** investigation, validation, methodology, project administration, writing – original draft, writing – review and editing, supervision. **Muhammad Abdullah Kashif:** methodology, project administration, writing – review and editing, supervision. **Soban Raza:** software, writing – review and editing, supervision. **Khawaja Haris Ismail:** visualization, writing – review and editing, supervision. **Javed Iqbal:** project administration, writing – review and editing, supervision. **Raheel Ahmed:** project administration, writing – review and editing, supervision. **Areeba Ahsan:** writing – review and editing, supervision. **Mushood Ahmed:** writing – review and editing, supervision.

## Ethics Statement

The authors have nothing to report.

## Consent

The authors have nothing to report.

## Conflicts of Interest

The authors declare no conflicts of interest.

## Supporting information

Supporting Information S1

## Data Availability

All data generated or analyzed during this study are included in this article. Further inquiries can be directed to the corresponding author.
